# Morphological and morphometric measurement of the temporomandibular joint of small and medium-weight dogs with different skull shapes

**DOI:** 10.3389/fvets.2024.1407761

**Published:** 2024-05-09

**Authors:** Ina Quadflieg, Holger A. Volk, Björn Sake, Benjamin Metje

**Affiliations:** ^1^Department of Small Animal Medicine and Surgery, University of Veterinary Medicine Hannover, Hannover, Germany; ^2^Institute for Animal Hygiene, Animal Welfare and Farm Animal Behavior, University of Veterinary Medicine Hannover, Hannover, Germany

**Keywords:** small animal, computed tomography, morphometry, temporomandibular joint, temporomandibular disorders

## Abstract

**Background:**

The recognition and diagnosis of canine temporomandibular joint (TMJ) disease can be a challenge, often leaving them undiagnosed. Although computed tomography (CT) has proved to be highly efficacious in detecting joint disease in the TMJ, morphometric and morphological studies of the normal TMJ have been scarce. Especially, skull type specific anatomical differences of the TMJ in dogs of different weights and skull morphologies have received limited attention.

**Objective:**

This study aimed to compare the TMJ morphologies of dogs across different weight classes and skull types.

**Study design:**

Retrospective study.

**Methods:**

CT scans were used to measure the depth and width of the Fossa mandibularis and two angles between the Fossa mandibularis and the Caput mandibulae in a total of 92 dogs and 182 mandibular joints, respectively.

**Results:**

The TMJ varied in terms of weight groups and skull indices. Shallow mandibular pits, underdeveloped retroarticular processes, and reduced joint congruency were observed particularly in light-weight and brachycephalic dogs. Conversely, dolichocephalic animals displayed deep joint pits, pronounced joint congruency, and a well-developed Processus retroarticularis.

**Main limitations:**

Observer learning curve; not every skull shape was represented in each weight group.

## Introduction

The canine temporomandibular joint (TMJ) is a bilateral synovial joint that takes an essential role in food ingestion and animal communication (1). It is formed by the Fossa mandibularis, which is part of the zygomatic process of the temporal bone, and the condyle of the mandible (Caput mandibulae). The TMJ can be divided into two separate articular cavities, that are subdivided by the Discus articularis. The upper part of the joint, which is bounded by the Fossa mandibularis of the Os temporalis and the Discus articularis, titled the discotemporal joint. The lower compartment of the joint extends from the Discus articularis to the Caput mandibulae and is called the discomandibular joint (2, 3).

Due to the complex anatomy of the carnivore skull, imaging of the TMJ often comes with significant challenges for veterinary surgeons (4, 5). Because of the better visualization of bony structures and the possibility of creating three-dimensional images, the examination of the TMJ utilizing computed tomography has been recommended (6, 7).

Primary TMJ disorders are currently rarely recognized in dogs, however, disorders affecting mastication and dental occlusion are common, which require evaluation of the joint (4). Therefore, it is essential to know the anatomical features of the TMJ of each dog breed and thus different skull types to define pathologic conditions (4, 5). The skull shape of the dog can be divided into three different skull types, which are called dolichocephalic, mesocephalic and brachycephalic (8). However, the dependence of skull shape in relation to the morphology of the articular surfaces of the TMJ of different dog breeds and weight classes has not yet been adequately studied (5).

Large breed variations have already been described in the literature in regard to the alignment of the TMJs (9). Most recently, the extent of breed disposition is illustrated by an additional publication, using a classification system for TMJs of various brachycephalic dog breeds (10).

Several other publications already indicate specific breed dispositions in relation to TMJ disorders, which in particular describe TMJ dysplasia (6). Poor joint congruency due to insufficiently developed joint cavities as well as joint processes can lead to a pronounced instability of the TMJ and can be accompanied by subluxations and luxations of the joint. These studies mainly concluded breed dispositions in French Bulldogs (10), Cavalier King Charles Spaniels (11), Dachshunds (12), American Cocker Spaniels (13), Basset Hounds (14, 15), Irish Setters (16), Boxers, Golden, and Labrador Retrievers (17).

In addition, a study published in 2016, which described the morphological appearance and congruence of the articular surfaces in different dog breeds using morphometric measurements, shows that especially the smaller dog breeds have pronounced TMJ incongruence (5). The study’s authors acknowledged that their sample size only included a few small dog breeds, which may limit the generalizability of the results to smaller dog breeds in general. Therefore, further investigation of small dog breeds is warranted. Moreover, a study was published in 2017 in which the TMJ ratios of different skull shapes and sizes were examined using geometric morphometric analyzes (18). The study came to the conclusion that there are significant differences between the various skull shapes and sizes. To verify this result and to determine the influence of skull shape and weight in dogs with a low to medium weight, the morphology of the TMJ was examined in this study by means of a further morphometric analysis.

The aim of the current (CT) based study was to investigate further differences in TMJ morphology in medium and small breed dogs with different cranial conformation and weight classes. This study will further improve the understanding of morphological differences of TMJs in relation to the dog’s signalment.

## Materials and methods

For this retrospective study, CT images of the skull from 07/2021 to 04/2023 of the Department of Small Animal Medicine and Surgery of the University of Veterinary Medicine Hannover, Germany, were reviewed. Only CT images of small to medium-sized dogs (<20 kg) and had no pre-report TMJ problems or pathology were used. All animals were weighed by clinical staff immediately before the CT examination. In total, 97 dogs (194 temporomandibular joints) were included in the study. Six animals (12 temporomandibular joints) had to be excluded from the study due to deficiencies in imaging or visible pathological alterations of the TMJ. Therefore a total of 182 TMJs from 91 different dogs were evaluated. There were 23 different breeds of dogs represented: Beagle (3), Bolonka Zwetna (1), Cavalier King Charles Spaniel (1), Chihuahua (4), Cocker Spaniel (3), Coton de Tulear (1), Dachshund (7), French Bulldog (12), Havanese (1), Maltese (3), Mini Australian Shepard (1), Crossbreed (17), Pug (5), Papillon (1), Pinscher (4), Podenco (1), Prague Rattler (1), Poodle (3), Sheltie (2), Shih Tzu (1), Silken Windsprite (1), Terrier (17), and Miniature Spitz (1). The age of the animals ranged from one to 15 years (median 7.9 years). Included were 45 females and 46 males. All CT scans were acquired with the same CT scanner (IQon spectral CT, Philips). The selected kilovoltage (KV) was 120 Kv in all CT scans. To accurately match the image quality to the skull size, the amount of charge flowing during the exposure in milliampere-seconds (mAs) was individually adjusted to the animal. The pitch ranged from 0.39 to 0.8 for all images used and the slice thickness was 1 (mm) in each case.

All patients were under general anesthesia and placed in sternal recumbency for the CT scan. The Rima oris was kept slightly open due to intubation. The lower jaw of the animals was always positioned horizontally, if necessary, a pillow was used for optional positioning under the lower jaw. All owners gave written consent that their data and images could be used for research. The study was approved by the local ethic and welfare committee.

The images were evaluated and measured using the DICOM processing program Horos (version 3.3) using a bone window (window level 300 and window width 1,500). First, all skulls were displayed in a sagittal and a dorsal section, and the skull index was determined for each animal (19). For this purpose, the length of the skull from the most cranial point of the Os incisivum to the most caudal point of the skull of the Os occipitale was measured in the sagittal sectional image. The dorsal section plane was used to measure the width of the skull. The longest distance starting from left to right Os zygomaticum was determined. From these measurement data, the cranial index was calculated as already used in other publications (19). All animals were then categorized into three different skull shapes: dolichocephalic, mesocephalic, and brachycephalic (8). Eight of the measured animals were assigned to the dolichocephalic cranial group, 38 dogs corresponded to the mesocephalic group, and 45 animals were categorized to the brachycephalic group. In addition, the animals were divided into four different weight categories in 5 kg increments: Group A: <5 kg, Group B: 5–10 kg, Group C: 11–15 kg, Group D: 16- < 20 kg. In this context, 14 animals were assigned to weight group A, 36 animals were assigned to weight group B, weight group C comprised 32 animals, followed by 9 animals in weight group D.

Subsequently, the image material used was utilized to make four different measurements per TMJ. First, anatomical landmarks were selected, which were recorded by default and after which, if necessary, a minimal axis adjustment was applied, which could be made in the three-dimensional reconstruction software. The alignment of the images to be measured was considered sufficient if the Caput mandibulae appeared symmetrical in the axial, transversal and coronal reconstruction, and the Crista nuchae was sufficiently visible in the sagittal reconstruction. These landmarks were also used for the subsequent TMJ measurements.

**Figure 1:**
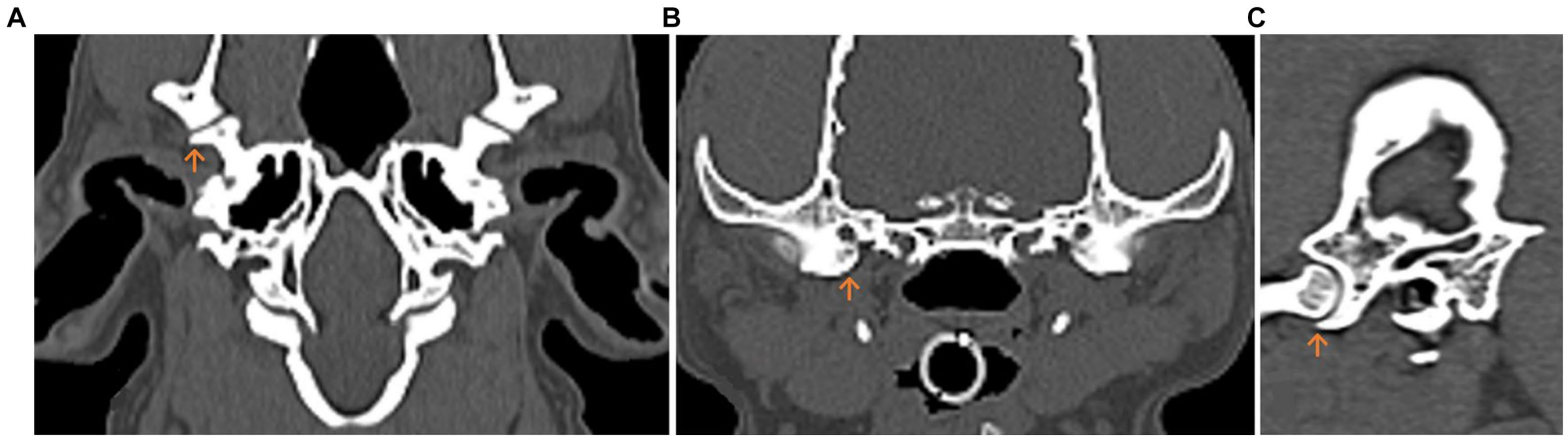
Multiplanar computed tomography (CT) reconstruction of the temporomandibular joint (TMJ) in the dog. The Processus retroarticularis (orange arrow) reaching maximal ventral extension is displayed. **(A)** Dorsal. **(B)** Transverse. **(C)** Sagittal planes.

These orientation points were selected in the sagittal sectional image at maximum congruence between the Fossa mandibularis and the Caput mandibulae, where the Processus retroarticularis was in maximum ventral alignment in respect to the Caput mandibulae (5) (Figure 1).

The following anatomical landmarks were evaluated on the sagittal section: the Fossa mandibularis, the maximum extent of the Processus retroarticularis, the dorsal eminence of the Fossa mandibularis, the Crista nuchae of the Os occipitale, and the estimated axis of rotation of the condylar process (EARCP). The dorsal prominence of the Fossa mandibularis as well as the EARPC are not listed in the Nomina Anatomica Veterinaria, but were mandatory for the following measurements (5, 20). The EARCP describes an applied circle on the condyle of the mandible (Caput mandibulae) in sagittal image reconstruction. With the listed program, the circle was carefully adapted to the given joint contours. The software also determined the center of the circle, which was essential for the angle measurements mentioned later. All previously mentioned landmarks were used for the subsequent measurements.

**Figure 2:**
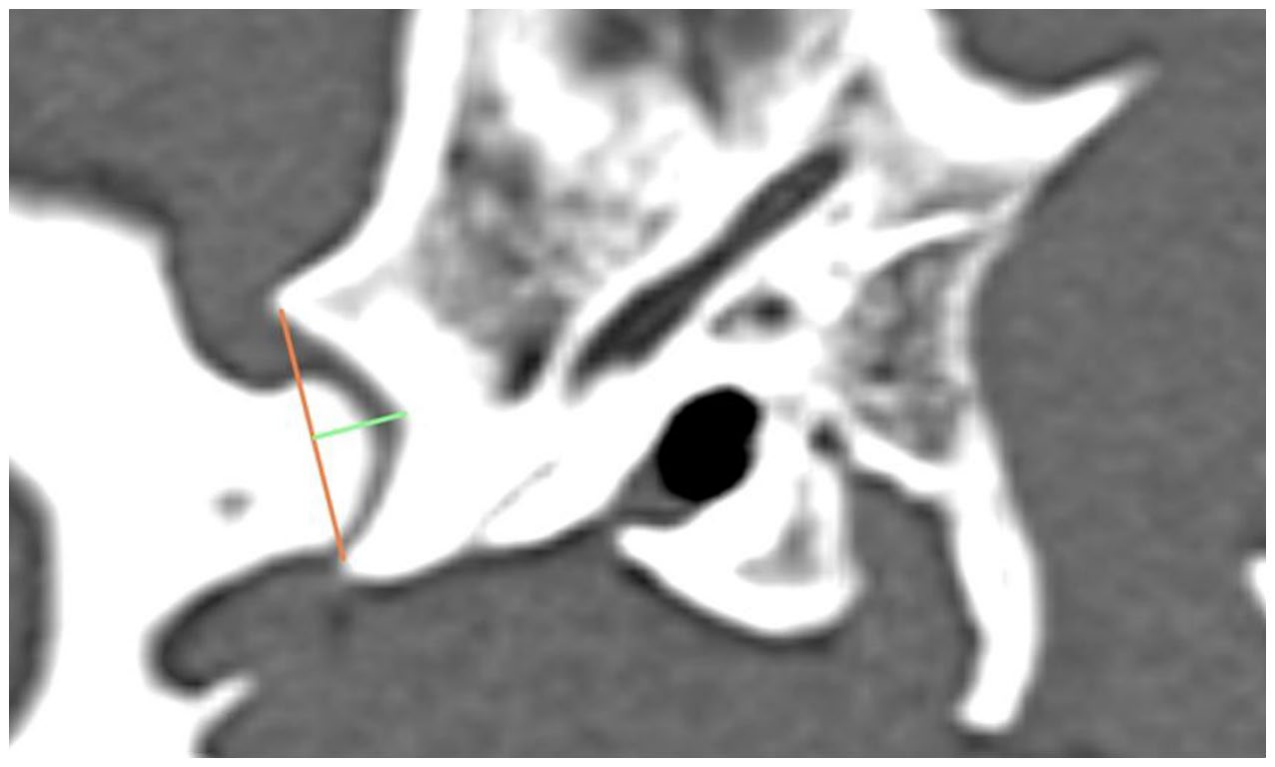
The graphic shows the lateral aspect of a dog’s skull in a sagittal computed tomography reconstruction, highlighting the width (orange line) and depth (green line) of the Fossa mandibularis.

The width and depth of the Fossa mandibularis were determined and the most ventral point of the Processus retroarticularis was selected. A measurement from this point to the dorsal eminence of the Fossa mandibularis was performed. The depth of the Fossa was determined by following a line down the midline from the previously drawn straight line to the deepest point of the subchondral bone of the Fossa (Figure 2). Subsequently, two angles were measured, which provide information about the congruency of the joint (angle 2) and show the extent of the Processus retroarticularis in relation to the Caput mandibulae (angle 1).

**Figure 3:**
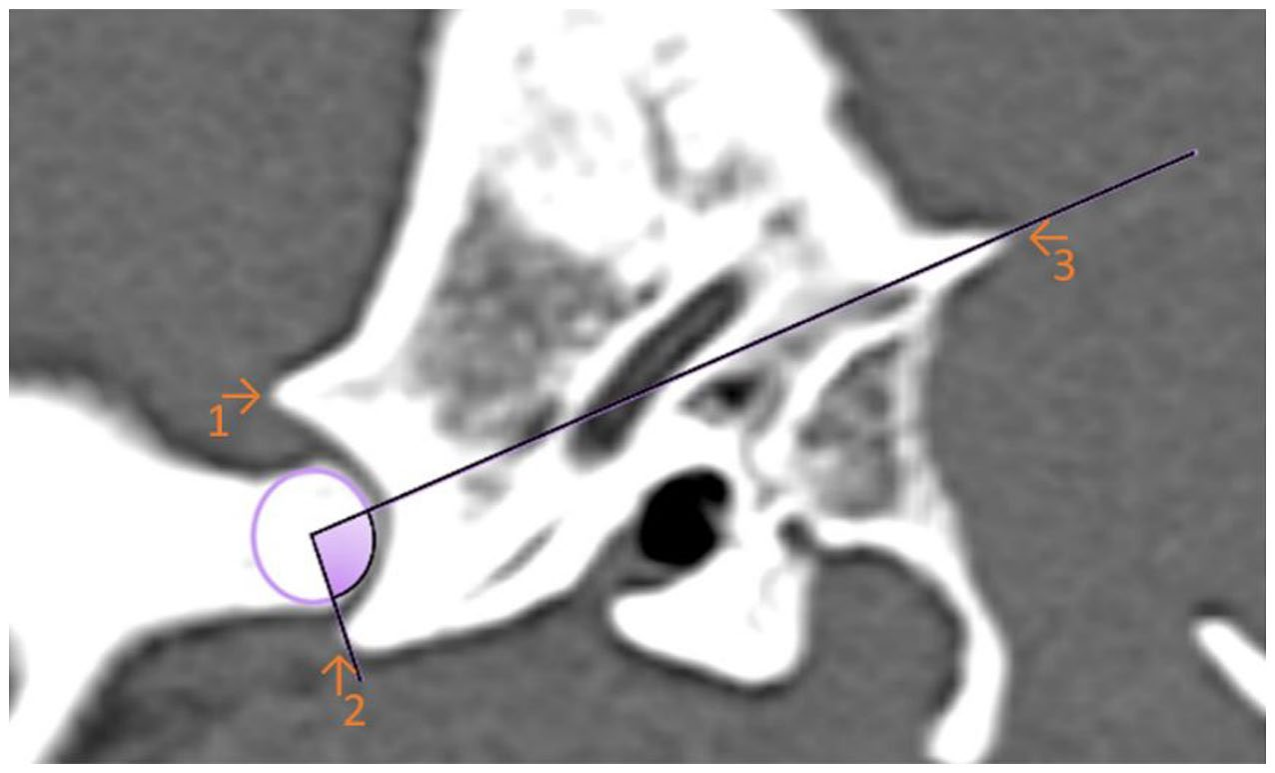
Sagittal computed tomography reconstruction image of a dog’s temporomandibular joint depicting angle 1. Angle 1 is formed by a line between the maximal ventral extension of the Processus retroarticularis and the Crista nuchae with the EARCP (purple circle). The filled (purple) area represents the measured angle. (1) Dorsal eminence of the Fossa mandibularis. (2) Processus retroarticularis. (3) Crista nuchae.

**Figure 4:**
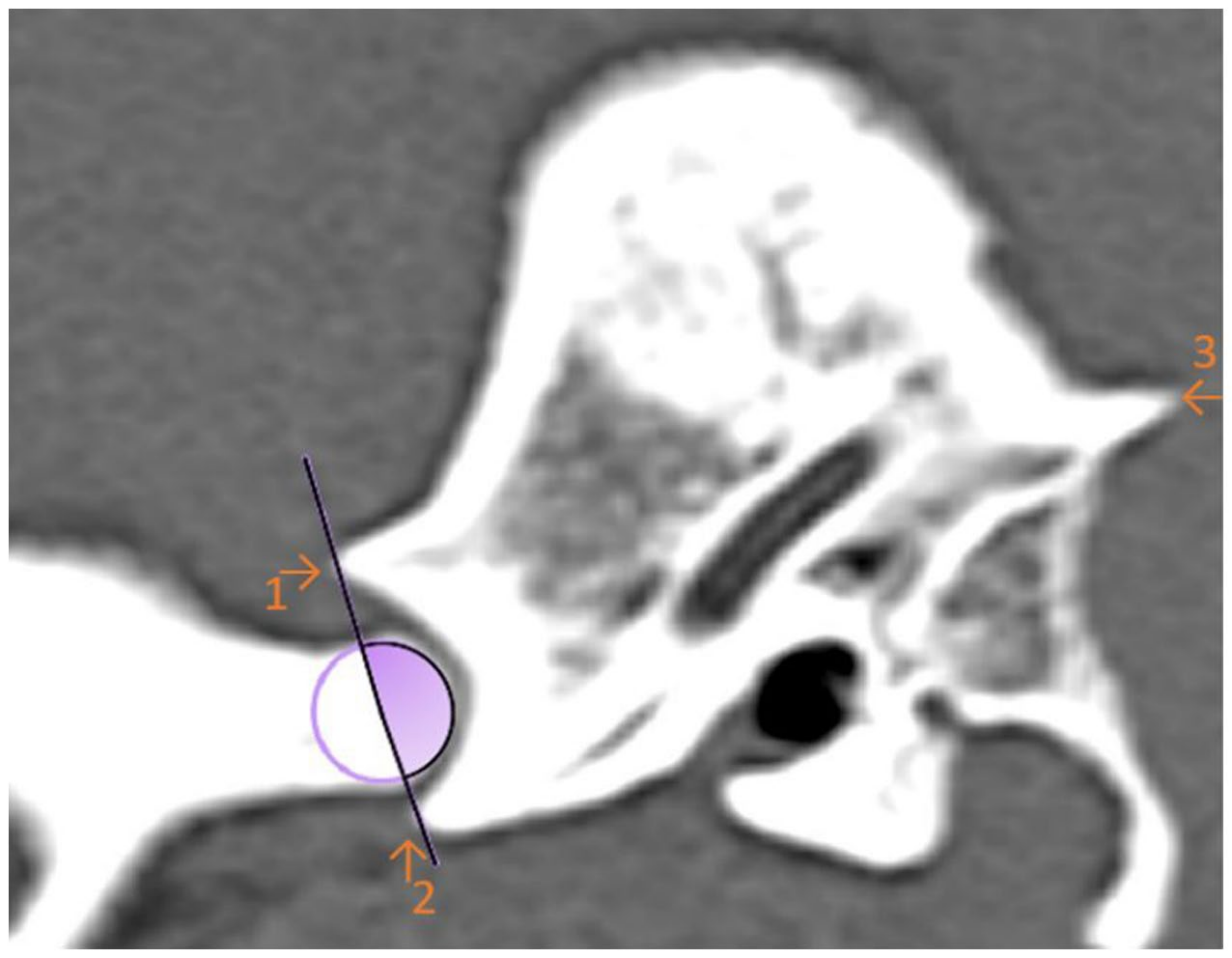
Sagittal computed tomography reconstruction image of a dog’s temporomandibular joint depicting angle 2. Angle 2 is formed by the linkage the maximal ventral extension of the Processus retroarticularis and the dorsal articular eminence of the mandibular fossa with the estimated axis of rotation of the condylar process (circle). The filled (purple) area represents the measured angle. (1) Dorsal eminence of the Fossa mandibularis. (2) Processus retroarticularis. (3) Crista nuchae.

For the first angle to be determined, the degree of the most ventral point of the Processus retroarticularis was measured over the previously selected EARCP of the respective condyle to the Crista nuchae (Figure 3). The angle resulting from the dorsal eminence of the Fossa mandibularis, over the EARCP and the most ventral point of the Processus retroarticularis was defined as angle 2 (Figure 4). All the above measurements were obtained in total three times by the same observer at different time periods.

### Statistical analysis

All determined values were entered into a suitable software [Microsoft Excel (Version 2,309) 2023]. Subsequently, the extracted data were transferred to SAS software (Enterprise Guide 7.15 and SAS software 9.4) and to the GraphPad Prism analysis program to be evaluated. For the graphic representation of the won values, the program GraphPad Prism was used likewise. An intra-observer comparison was performed using the intraclass correlation coefficient (ICC) to identify measurement deviations of the determined three measurements per variable. Following this, the arithmetic mean was formed from these three measurements, which was used for the statistical evaluation. Thereafter, a skull index-oriented data analysis as well as a weight classified data analysis was performed to investigate a possible correlation of the respective parameter with the determined TMJ values. In addition, an evaluation of the relationship between the width and depth of the Fossa mandibularis was carried out. For this purpose, the two values were divided with each other, and the quotient determined was used for the evaluation. A gender-specific analysis was also performed.

A descriptive data analysis was conducted. The Shapiro–Wilk test was then used to test the normal distribution. A simple ANOVA was carried out to determine the significance of the data. As the data in each data set was not always normally distributed, different post-hoc tests were undertaken depending on the distribution pattern. For non-normally distributed data sets, the Kruskal-Wallis test was performed, followed by Dunn’s multiple comparison test. If the data was normally distributed, the Tukey multiple comparison test was used. Since the data sets were normally distributed for the evaluation of possible measurement discrepancies between the left and right temporomandibular joint of the animals, a t-test was performed. A *p*-value of <0.05 was considered significant.

## Results

**Figure 5:**
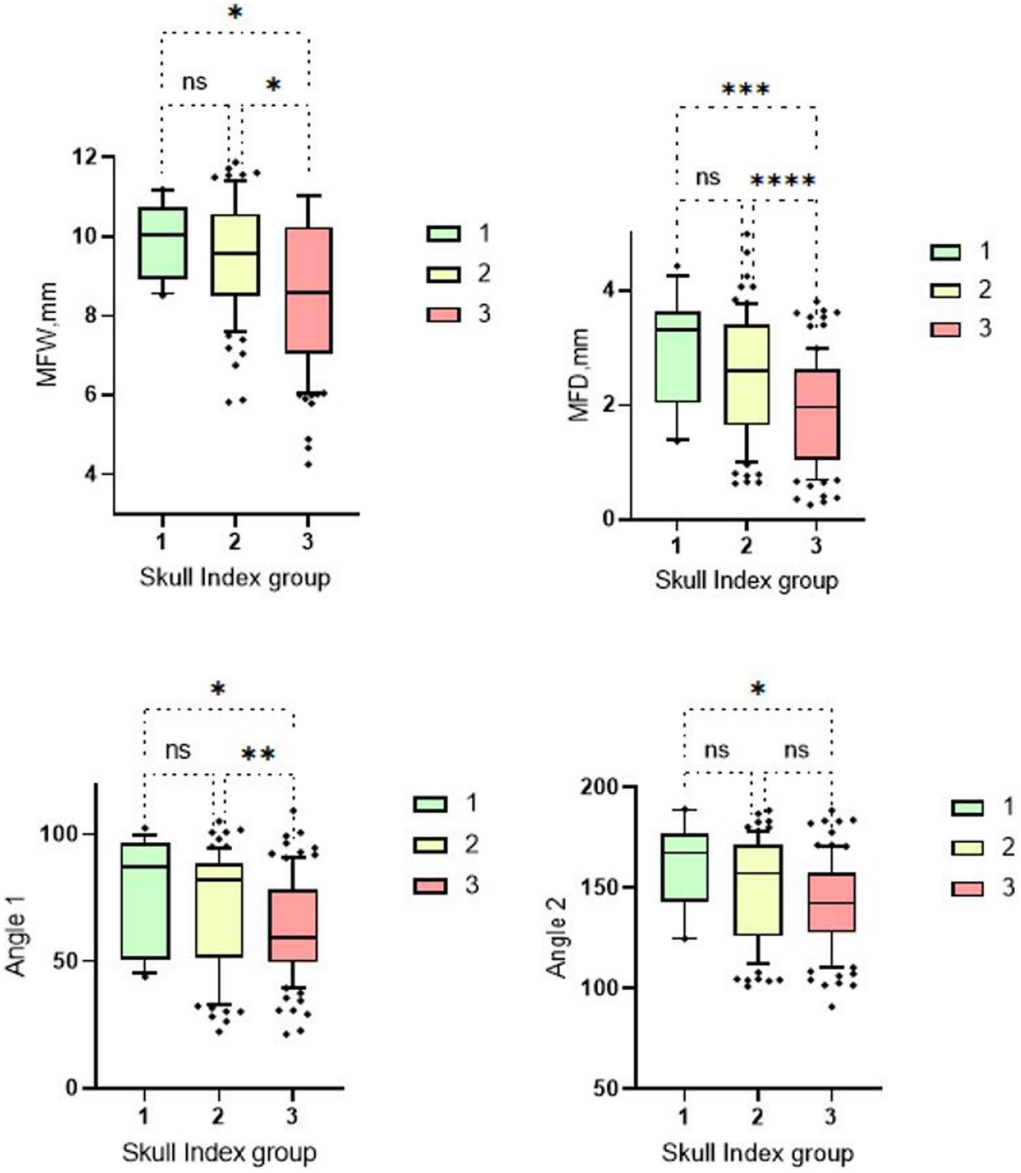
Box and whisker plots of Skull Index groups. The four box plots show the evaluation of the temporomandibular joint measurements carried out in relation to the three different skull shapes mentioned. Group 1: dolichocephalic dogs; Group 2: mesocephalic dogs; Group 3: brachycephalic dogs. Significant differences between the analyzed groups were detected in the analyzed data set. The significance was characterized by asterisks and connecting lines between the study groups. ^ns^*p* > 0.05; *p < 0.05; **p < 0.01; ***p < 0.001; ****p < 0.0001. Significant differences were found between the first and third cranial index groups for all measurement parameters (MWF: *p* = 0.04; MWD: *p* = 0.0005; Angle 1: *p* = 0.022; Angle 2: *p* = 0.01). Further significant differences can be seen between cranial index groups 2 and 3. Here, the measurements of the mandibular fossa (*p* = <0.0001–0.01) and angle 1 (*p* = 0.009) are significantly different. No significant differences were found between measurement groups 1 and 2.

The aim of this study was to compare dogs with different cranial morphologies and weight classes based on their morphometric TMJ data. In the three skull groups analyzed, Group 1 (dolichocephalic dogs) showed greater median widths and depths of the Fossa mandibularis than the mesocephalic and brachycephalic groups. The two measured angles were also larger in the dolichocephalic animals than in the comparison groups (Figure 5).

The brachycephalic animals had the lowest measured values. There were significant differences between skull group 1 and 3 for all four measurement parameters. When measuring the fossa width and depth and the angle 1, a significant difference was found between skull group 2 and skull group 3. Only between skull group 1 and 2 no significant differences were found (Figure 5).

**Figure 6:**
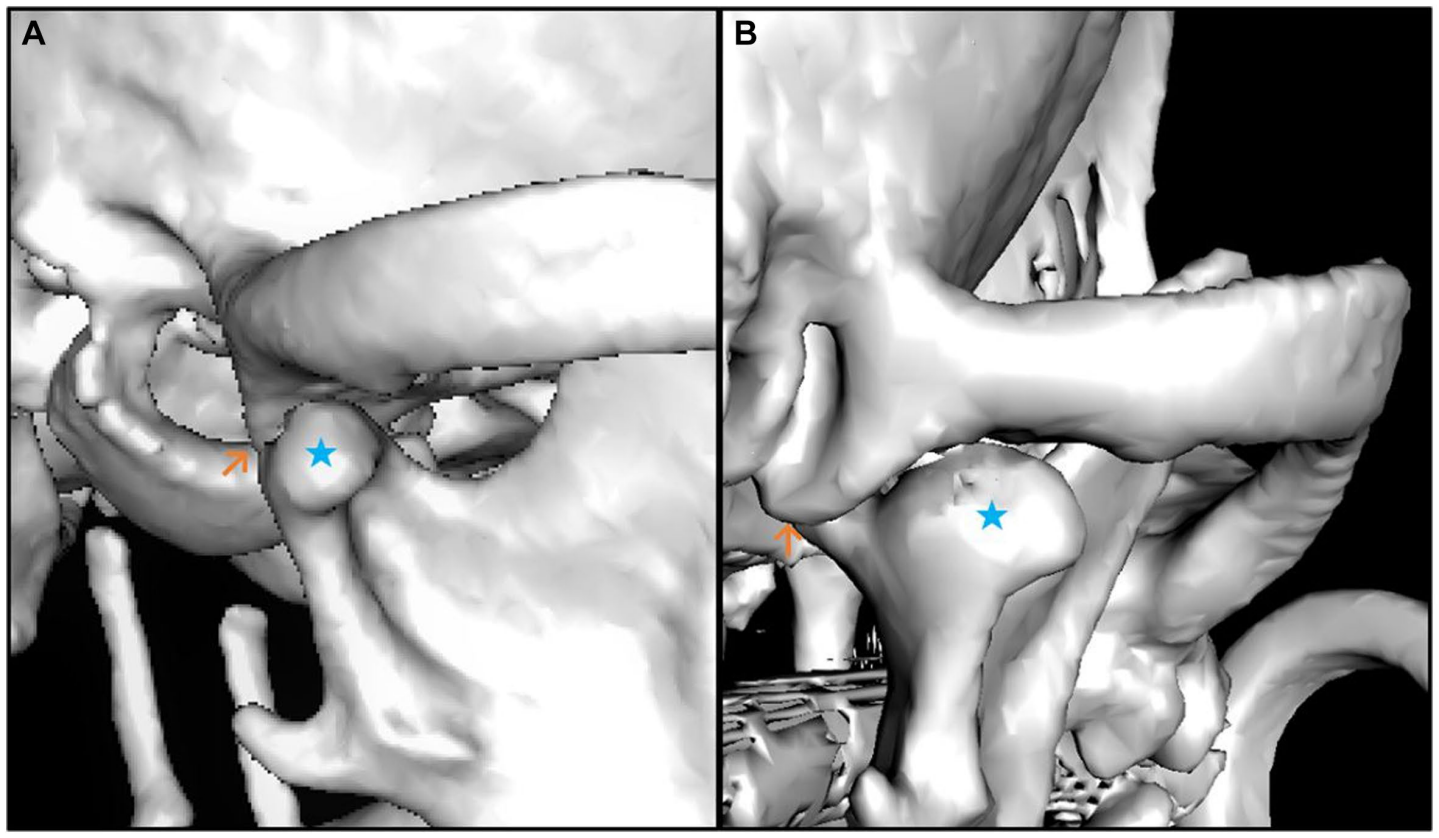
3D reconstruction of a brachycephalic dog skull showing minimal values in the evaluation of the measured parameters. The images show lateral **(A)** and caudal **(B)** views of the right temporomandibular joint. The caput mandibulae is highlighted with a blue star in both views. The Processus retroarticularis is marked (orange arrow) at the position where it reaches maximal ventral extension. The lateral view **(A)** shows that the Caput mandibulae is sparse limited by the Processus retroarticularis only on the medial side of the joint. The caudal view **(B)** shows that the Processus retroarticularis is nearly absent.

**Figure 7:**
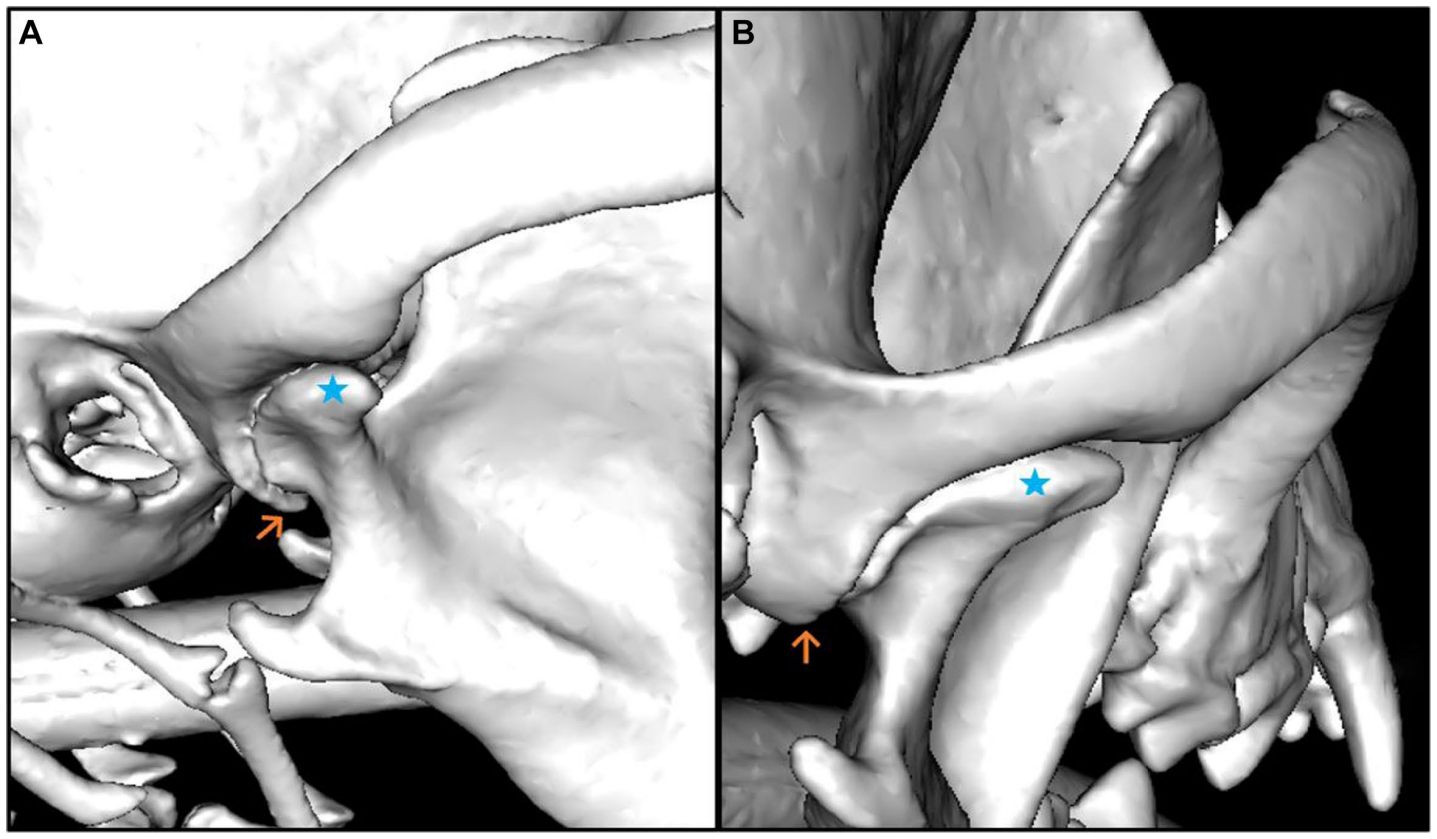
3D reconstruction of a dog skull with a dolichocephalic skull type, which shows maximum values in the evaluation of the measured parameters. The lateral **(A)** and caudal **(B)** views of the right temporomandibular joint are shown. The Caput mandibulae is marked with a blue star in both views. The Processus retroarticularis is marked (orange arrow) at the position where it reaches maximal ventral extension. It can be seen in the illustrations that the Processus retroarticularis surrounds the Caput mandibulae ventrally **(A)** and thus borders the joint cavity caudally **(B)**.

The median smallest width (4.25 mm) and the smallest depth (0.26 mm) of the mandibular fossa were found in the Chihuahua, and in these brachycephalic animals the articular surface presented as an almost straight surface (Figures 6, 7).

The median smallest angle one (21.27°) and the smallest angle two (90.99°) were also found in a brachycephalic animal, a French Bulldog. This animal had a missing *Processus retroarticularis.*

In this context, the different representation of the TMJ, the various cranial index groups, in the respective CT reconstruction was notable. The lower the value of the two angle sizes, the smaller or even absent the Processus retroarticularis appeared. In these cases, the condyle was thus not or insufficiently framed caudally. The assorted sizes of the fossa width and depth are also presented differently in the CT images, respectively. Animals exhibiting high values for these measurement variables displayed symmetrical, round-shaped articular cavities. In contrast, animals showcasing low values had asymmetrical, irregular, and almost straight joint shapes.

**Figure 8:**
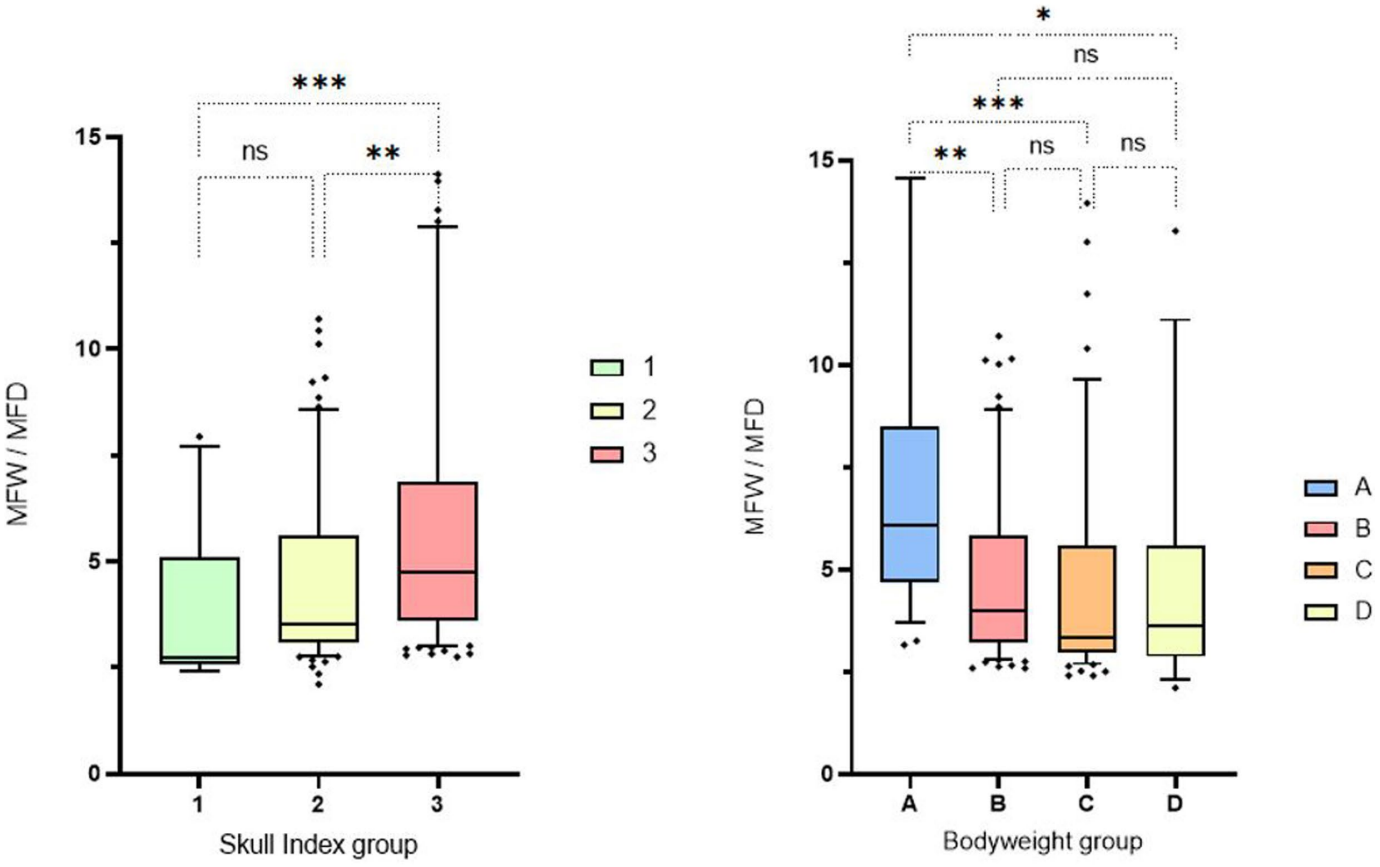
Box and whisker plots of Skull Index groups and Bodyweight groups. The three boxplots show the evaluation of the measurement of the fossa width/fossa depth, which was carried out in relation to the three different skull shapes mentioned (left side). The fossa width/fossa depth quotient of the 4 boxplots in relation to the weight division (right side). Significant differences between the analyzed groups were detected in the analyzed data set. The significance was characterized by asterisks and connecting lines between the study groups. ^ns^*p* > 0.05; *p < 0.05; **p < 0.01; ***p < 0.001; ****p < 0.0001. Significant differences were found between cranial index groups 1 and 3 (*p* = 0.0006) and between cranial index groups 2 and 3 (*p* = 0.0017). Significance could also be determined in the weight-associated analysis. Remarkable differences were found between weight group 1 and the other three comparison groups, with significance values ranging from p = 0.001 to *p* = 0.015.

This visual observation was confirmed mathematically when analyzing the quotient calculated from the fossa width/fossa depth. Significant differences were found between the individual study groups. In particular, dolichocephalic animals had significantly lower median values when calculating the quotient. These animals therefore have a deeper and narrower fossa, which is also more congruent in shape. Brachycephalic animals in particular show high values, which indicate a flat and broad fossa. A tendency can also be recognized in the weight-related assessment: Especially lightweight dogs (<5 kg) have flatter and wider fossas (Figure 8).

**Figure 9:**
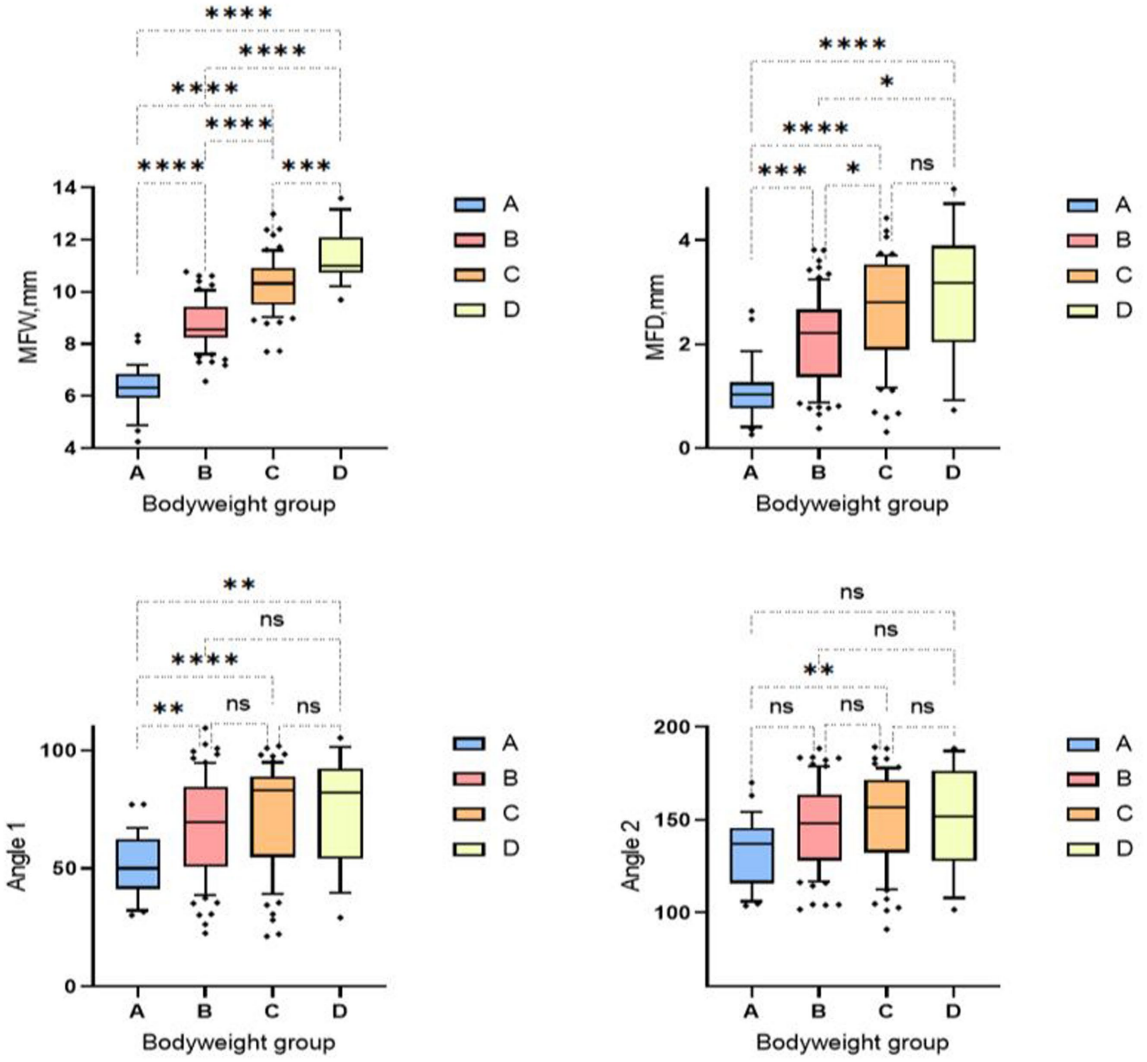
Box and whisker plots of the bodyweight groups. Group 1: < 5 kg; Group 2: 6–10 kg; Group 3: 11–15 kg; Group 4: 16–20 kg. The four box plots show the evaluation of the temporomandibular joint measurements carried out in relation to the four bodyweight groups. Significant differences between the analyzed groups were detected in the analyzed data set. The significance was characterized by asterisks and connecting lines between the study groups. ^ns^*p* > 0.05; *p < 0.05; **p < 0.01; ***p < 0.001; ****p < 0.0001. Significant differences between the analyzed groups were detected in the analyzed data set. Fossa width showed significant differences between all weight groups (*p* = 0.0001–< 0.0001). Fossa depth also showed significant differences between all weight groups (*p* = <0.0001–0.04), except for weight group C, which showed no significant differences from weight group D. The first measured angle showed significances between weight group A in relation to all three other weight groups (*p* = <0.001–0.006). Significant readings were documented between weight group A and weight group C for measurement angle two (*p* = 0.004).

Animals in weight group A showed smaller median mandibular fossa width and mandibular fossa depth and angle measurements than weight groups B-D. Weight group B again showed smaller results of the four measurement variables than weight groups C and D. It is noticeable that in weight group D, only two measured values assumed larger values in the course than in group C. The measured angle one provides information about the extent of the Processus retroarticularis and showed values that were 0.82° lower in weight group D than in weight group C. Angle two, which provides information about joint congruency, showed an average value 4.86° lower than the heard angle two of weight group C (Figure 9). No significant difference was found between males and females in relation to the four different study variables when the weight groups and skull index groups were analyzed.

The intra-observer comparisons via ICC performed, for mandibular fossa width (ICC = 0.94), mandibular fossa depth (ICC = 0.97), and angle measurements one (ICC = 0.97) and angle measurement two (ICC = 0.96), were excellent. The repetitive skull length readings (ICC = 0.99) and skull width dimensions (ICC = 0.99), most consistent. As the comparative descriptive analysis and paired *t*-test of the left and right TMJ sides with a defined significance level of *p* < 0.05 showed no significant difference in all determinations performed, the TMJ measurements were subsequently evaluated together.

## Discussion

This study describes the morphometric measurement of the TMJ of dogs using CT and provides information on the morphological characteristics of the TMJ of dogs under 20 kg. The measured parameters of the different cranial index and weight groups show significant differences in the anatomical constitution of the Fossa mandibularis of these animals and equally significant differences in the congruence of the joint and the development of the Processus retroarticularis.

The canine TMJ has only been studied to a limited extent. This study builds on three previous studies in which morphometric measurements of the canine TMJ were performed (5, 10, 18). In two of the above studies, different breeds of brachycephalic and mesocephalic dogs were analyzed and it was shown that there are breed differences in the congruency of the TMJ, the arrangement of the retroarticular process and the shape of the mandibular fossa. However, these studies were limited to breed-specific evaluations (5, 10). Furthermore, the 2016 study acknowledges that the group size of low- and medium-weight dogs was not sufficiently investigated in this study.

This study thus follows on from a study conducted in 2017, which looked at the specific effects of different skull shapes and sizes on the temporomandibular joint in dogs (18). Also significant differences between the various skull shapes and sizes were demonstrated. Here too, light and short-headed dogs in particular showed poorer measurement results than long-headed or heavier dogs. In this context, the results obtained in the aforementioned study can be reconfirmed by the present study. However, given the nature of this study focusing on geometric 3D measurements without a designated classification of skull classes, and lacking documentation of the pathological impacts on the involved animals, we conducted a morphometric CT measurement test to explore this hypothesis further. Furthermore, studies have shown that particularly small dog breeds and also brachycephalic dog breeds are more frequently affected by orofacial and periodontal diseases than heavy or long-headed dogs (21–23). In this context, the question emerges regarding whether this phenomenon causes a change in TMJ configuration owing to altered skull alignment. A targeted investigation into these small dog types was carried out to address this research question. As in two of the previous studies, this study also showed that the Cavalier King Charles Spaniel, the Dachshund, and the French Bulldog had particularly low values for the respective TMJ measurements. These results support the hypothesis of an existing breed predisposition in these dog breeds (10–12). The French Bulldog was especially different because of its particularly wide and extremely flat joint cavities. The two angle measurements were also generally demonstrated to be lower in these animals. This result differs from the 2017 study, in which French Bulldogs had very diverse jaw joint shapes and therefore did not exclusively show low measurement results, as determined in this study. However, the precise number of French Bulldogs included in the 2017 study is lacking, potentially limiting the significance of this finding and also complicating comparability with other existing studies. In order to put these results into context, a follow-up study should be carried out to investigate the specific shape and variation of the jaw joint of this breed.

In contrast to the results of the TMJ measurements of the 2016 and 2023 studies, the Shih Tzu and Pug breeds in the present study did not present exceptionally poor measurements in relation to the four different measurement parameters (5, 10). However, it should be noted that the focus of this study is not on the evaluation of breed-specific predispositions and thus only a limited number of animals per dog breed is available, which may represent a limitation of this breed-specific result.

When the animals were analyzed according to the cranial index, significantly flatter and smaller Fossae mandibularis were found in brachycephalic dogs than in meso- and dolichocephalic dogs, which also correlated with significantly smaller angular measurements. The selected angle measurements provide information about the congruence and shape of the joint cavity and the Processus retroarticularis. However, it was also found that the fossa width/fossa depth quotient was significantly greater in brachycephalic animals than in meso- or dolichocephalic animals. These results thus indicate an irregularly shaped articular fossa and a little to underdeveloped Processus retroarticularis, so a reduced TMJ congruence can be assumed in brachycephalic animals. As already described in other studies, an underdeveloped to insufficiently developed Processus retroarticularis and an incongruent articular surface harbor an increased risk of TMJ pathologies, which can be associated with luxations, temporomandibular joint instability and dysplasia, for example (9, 24). A central problem here is that congenital TMJ defects and acquired pathologies are often not diagnosed. Clinical signs in animals with TMJ pathologies can vary widely or even be absent, making it exceedingly difficult for the treating veterinarian to recognize and interpret these symptoms after a general clinical examination (25). Further research is necessary to investigate the clinical presentation and diagnostic tools used to recognize TMJ disorders.

Dolichocephalic dogs showed both the widest and deepest Fossa mandibulares as well as a more congruent Fossa mandibularis and a more pronounced Processus retroarticularis, indicating an intact and stable developed joint. Looking at the original skull and TMJ shape of the dog’s evolutionary ancestors, there are no major differences in wolves concerning these features (26). The wolf’s TMJ is analogous to the well-integrated joint found in dolichocephalic dogs. This stability and functionality are critical for the wolf’s survival as a successful predator and food provider. Dog breeds that are now primarily bred for social purposes and for a certain visual appearance and no longer rely on hunting show distinct morphologies in their cranial joints and TMJ (18).

Despite the significant results of this study about the cranial morphology of different dogs, it should be mentioned that brachycephalic and mesocephalic animals do not necessarily have worse values regarding the TMJ measurements than dogs with a dolichocephalic head shape. In some cases, similar values were measured in the above-mentioned head shapes as in the dolichocephalic animals. To conclude, it can be said that the skull shape is not necessarily associated with a specific TMJ morphology, although a clear tendency of the different skull groups can be observed (see Figure 5).

Previous studies on the measurement of dog skulls and determination of the skull index have resulted in specific categorizations of the various dog breeds into different skull types (brachy-, meso-, dolichocephalic) (8, 19, 27). In this study, by measuring the skull index of each individual dog, an adequate and precise categorization could be made into the three different skull shapes, regardless of breed affiliation. This concept proved to be extremely useful, as it was found that there can be considerable variation in skull morphology within a dog breed. Therefore, animals of the same breed cannot necessarily be assigned to the same skull group (brachy-, meso-, dolichocephalic). An individual assessment and categorization of each animal into the corresponding skull group is therefore advisable. In addition, mixed breeds can also be assigned to a specific skull group by this method.

Our weight-specific analysis shows that lighter and therefore smaller dogs have smaller, and flatter joint cavities compared to heavier and larger dogs. Significantly lower measured values were also found for the two measured angles. Similarly, the wide fossa/deep fossa quotient showed significantly higher values in weight group A (<5 kg), and it can therefore be assumed that smaller dogs have a less pronounced Processus retroarticularis and incongruent joint cavities due to lower measured values and a higher quotient (see Figure 9). However, this assumption does not apply to the analyzed weight group D (16–20 kg), in which only the measurements of the depth and width of the Fossa mandibularis showed larger values than in the previous weight classes. There is a distinctive feature in this group regarding the angular parameters: The measured values are lower than in the previous weight group C (11–15 kg). It can therefore be concluded that, on average, animals in this weight group do not have improved mandibular joint congruence or larger Processus retroarticularis compared to the lower weight class (11–15 kg). These results can be explained by the animals included in this weight class. Four French Bulldogs were measured in this group, and all of them showed conspicuously low angular measurement results. Due to the small number of animals and the limited breed diversity in this weight class (*n* = 9), it is important to critically evaluate these results. Further studies with a higher number and more breed diversity in this weight group are required to verify the deterioration in jaw joint congruence and lower expression of the Processus retroarticularis observed in this study.

Another important aspect of this study is the weight classification of the animals. All animals were weighed prior to CT examination. However, the body condition score was unfortunately not consistently determined and can therefore not considered in the current study due to the study’s retrospective nature. Increased body condition scores secondary to obesity could have biased the results, as only weight was considered in the study. Consideration of the body condition score could be taken into account in a follow-up study to ensure a more precise classification of animals into the correct weight class and minimize this potential bias.

To ensure safe ventilation during the CT examination of the animals, all animals were intubated, resulting in a slight opening of the mouth. Angle measurements 1 and 2 utilize different anatomical landmarks from two distinct cranial bones (mandible and maxilla) for measurement. Due to the mobility of these cranial bones relative to each other, the question arises as to whether the degree of mouth opening can influence the angle measurements. Given the anatomy of the temporomandibular joint in dogs and cats, which mainly allows for the opening and closing of the mouth with minimal forward, backwards, or sideways movement, it is assumed that the center of the caput mandibulae, used for the mentioned measurements, remains constant regardless of the mandible’s axis of rotation and that potential sources of error can be excluded through these measurements (28).

Furthermore, the animals were uniformly and symmetrically positioned during the CT examination to achieve comparable results. To verify this assumption, a comparative morphometric CT study could be conducted on non-intubated animals or cadavers.

A gender-related evaluation of the cranial index and weight groups was also carried out. However, no significant differences were found between the sexes. According to this results gender has no additional influence on the morphology of the TMJ.

Despite the limitation that not all three cranial index groups to be examined were present in all weight classes and that the number of animals per group also differed, this study showed objective differences in the anatomical expression of the TMJs of these various groups. To investigate the level of intra-observer bias and the accuracy of the measurement results of the three measurement cycles performed, the intra-observer adjustment was used. This test showed a high degree of reliability and reproducibility of the measurement methodology.

Certainly, it should be pointed out that due to the existing diverse joint anatomy of the dogs, the structures to be measured can be represented differently in the image and this can therefore present a challenge when taking measurements. By implementing a uniform methodology the influence to the measurements was minimized and therefore it can be concluded that brachycephalic dogs as a whole show worse measurement values than meso- and dolichocephalic animals.

Many studies before have shown that especially brachycephalic dogs’ morphology can impact their quality of life because of their breeding standard (29, 30). In addition to the fact that these animals not only show increased respiratory problems (31), dermatological diseases (32, 33) and other significant body changes (34, 35), it has now been confirmed that the TMJs are also affected negatively by their breeding, which is associated with a significantly shorter and wider skull morphology. Therefore, the evaluation of the TMJ in these predisposed dogs should be considered when breeding and also when investigating their eating behavior. This study confirms the assumption of skull index specific TMJ morphology. Here, for the first time, TMJ measurements are made and compared for all three skull types. A weight-associated TMJ morphology was confirmed.

## Data availability statement

The raw data supporting the conclusions of this article will be made available by the authors on request, without undue reservation.

## Ethics statement

This study was conducted according to the ethical standards of the University of Veterinary Medicine Hannover Foundation. The doctoral thesis committee of the university, which acts as the university’s ethics committee, validated the project in accordance with the ethical guidelines regarding research with retrospective datas and approved this study. The data protection officer reviewed the proposed project regarding the observance of the data protection law and gave per-mission to perform this study. The studies were conducted in accordance with the local legislation and institutional requirements. Written informed consent was obtained from the owners for the participation of their animals in this study.

## Author contributions

IQ: Conceptualization, Data curation, Formal analysis, Investigation, Methodology, Software, Validation, Visualization, Writing – original draft, Writing – review & editing, Resources. HV: Conceptualization, Investigation, Methodology, Project administration, Supervision, Validation, Visualization, Writing – review & editing. BS: Formal analysis, Writing – review & editing. BM: Conceptualization, Investigation, Methodology, Project administration, Supervision, Validation, Visualization, Writing – review & editing.
