# Supplementary material for: Morphological and morphometric measurement of the temporomandibular joint of small and medium-weight dogs with different skull shapes
Source: Front Vet Sci. 2024 May 9;11:1407761. doi: 10.3389/fvets.2024.1407761 (PMC11112034; doi:10.3389/fvets.2024.1407761)
Supplement: Supplementary file 1 [file Data_Sheet_1.docx]

Supplementary Material

# Supplementary Data

Table 1. Representation of the dogs included in this study divided by weight groups (A-D) and skull Index Groups (1-3).

Abbreviations: Group A: <5kg, Group B: 5-10kg, Group C: 10-15kg, Group D: 15-20kg

Group 1: dolichocephalic dogs; Group 2: mesocephalic dogs; Group 3: brachycephalic dogs

| Weight/Skull Index | A | B | C | D |
| --- | --- | --- | --- | --- |
| 1 | 0 | 3 | 5 | 0 |
| 2 | 2 | 15 | 16 | 5 |
| 3 | 12 | 18 | 11 | 4 |

Table 2. Maximum and Minimum Values with respective Medians of TMJ measurement in the three different head morphologies

Abbreviations: MFD, depth of the mandibular fossa; MDW, width of the mandibular Fossa.

|  | Skull Index 1 | Skull Index 2 | Skull Index 3 |
| --- | --- | --- | --- |
| MFW, mm | 8.51-11.19 (10.04) | 5.82-12.08 (9.57) | 4.25-13.59 (8.58) |
| MFD, mm | 1.37-4.43 (3.32) | 0.63-4.99 (2.61) | 0.26-3.81 (1.96) |
| Angle 1 | 43.95-102.50 (87.31) | 22.23-105.26 (82.21) | 21.27-109.44 (59.40) |
| Angle 2 | 124.81-189.15 (167.43) | 101.07-188.55 (157.14) | 90.99-188.54 (142.30) |

Table 3. Maximum and Minimum Values with respective medians of TMJ measurement in the four different weight groups (A-D)

Abbreviation: MFD, depth of the mandibular fossa; MDW, width of the mandibular Fossa.

|  | Weight Group A | Weight group B | Weight group C | Weight group D |
| --- | --- | --- | --- | --- |
| MFW, mm | 4.25- 8.33 (6.30) | 6.56-10.77 (8.56) | 7.7-12.99 (10.31) | 9.70-13.59 (11) |
| MFD, mm | 0.26-2.64 (1.03) | 0.38-3.82 (2.2) | 0.31-4.43 (2.8) | 0.73-4.99 (3.18) |
| Angle 1 | 30.19-77.17 (50.03) | 22.60-109.43 (69.66) | 21.27-101.84 (82.89) | 29.15-105.26 (82.07) |
| Angle 2 | 103.69-170.06 (136.78) | 101.65-188.54 (147.90) | 90.99-189.15 (156.63) | 101.56-188.55 (151.77) |

Figure 1. Box and whisker plots of the skull index groups.

The four box plots show the evaluation of the temporomandibular joint measurements, which were carried out in relation to the three skull shapes mentioned and as a function of gender.

Group 1: dolichocephalic dogs; group 2: mesocephalic dogs; group 3: brachycephalic dogs; w: female, m: male

No significant differences (ns: p> 0.05) were found between the groups studied in the data set analysed.

Figure 2. Box and whisker plots of the bodyweight groups.

Group 1: < 5 kg; Group 2: 6-10 kg; Group 3: 11-15 kg; Group 4: 16-20 kg; w: female, m: male

The four box plots show the evaluation of the temporomandibular joint measurements carried out in relation to the four bodyweight groups and as a function of gender. No significant differences (ns: p> 0.05) were found between the groups studied in the data set analysed.
